# Efficacy of Anti-Vascular Endothelial Growth Factor (VEGF) Therapy for Age-Related Macular Degeneration

**DOI:** 10.7759/cureus.74776

**Published:** 2024-11-29

**Authors:** Saad Bidiwala

**Affiliations:** 1 General Medicine, Barts Health National Health Service (NHS) Trust, London, GBR

**Keywords:** age-related macular degeneration (amd), amd, anti-vegf therapy, benefits of anti-vegf therapy, management of cnv in amd

## Abstract

Anti-vascular endothelial growth factor (VEGF) drugs are used for various diseases with abnormal proliferation of blood vessels. The use of these drugs in wet age-related macular degeneration (AMD) has proven to be highly effective. Various factors contribute to the efficacy of these drugs in different settings. Many studies have proven that these drugs effectively slow disease progress and improve visual outcomes. Factors contributing to the success or failure of the treatment include the genetic makeup of the patient, comorbidities, compliance with the clinic visits and injections, long-term follow-up for the treatment, socioeconomic status, and availability of different drugs. The treatment of choroidal neovascularization (CNV) in neovascular age-related macular degeneration (nAMD) has been revolutionized after the introduction of anti-VEGF therapy. However, there are still some gaps in the literature that require the attention of the researchers. Our literature review has evaluated anti-VEGF use over the years and analyzed the efficiency of drugs in different settings. It showed that all the anti-VEGF drugs depict similar visual results for one to two years. The long-term evaluation of any drug cannot be commented on yet and needs further evidence through different research. These drugs improve visual function and the anatomical results of patients with other eye problems. These drugs' adverse effects are rare but still an important point requiring further research. Clinical outcomes of the drugs must be ascertained through patients' eyes to assess the quality of life improvement appropriately. The cost-effectiveness of the drugs is a substantial debatable topic, as bevacizumab is cost-effective but still requires Food and Drug Administration (FDA) approval.

## Introduction and background

Age-related macular degeneration (AMD) is the leading cause of irreversible vision loss all over the world, especially in the developed world. Macular degeneration affects sharp vision and causes blindness, where the best corrected visual acuity (BCVA) is ≤20/200 in the better eye. AMD presents in two forms: dry and wet. The dry form of the degeneration is present in 80% of the forms, which consists of atrophy of the retinal pigment, retinal drusen, and hyperpigmentation of the retinal pigment epithelium. The remaining 20% of the patients suffer from the neovascular or wet type of AMD. There is a vascular generation background behind this type of disease development. The wet type is associated with choroidal neovascularization (CNV), which manifests as subretinal, intraretinal fluid collection, or hemorrhage. The prevalence of AMD is rising due to an increase in life expectancy and early detection of the disease. The physical structure of these vessels is abnormal, which causes leakage in these forces [1].

The previous treatments of CNV in AMD included laser photocoagulation and verteporfin-mediated photodynamic therapy (PDT), sometimes with intravitreal injections of triamcinolone. After the inculcation of anti-vascular endothelial growth factor (VEGF) therapy for CNV, the visual benefits between the older therapies and anti-VEGF therapy were compared [2]. The anti-VEGF therapy was better regarding visual standards than the older therapies. Still, these therapies are used in scenarios where patients resist the anti-VEGF agents. 

The anti-VEGFs are associated with the blockage of new vessels in any body part. VEGF therapy is associated with the blockage of proteinaceous growth factors that are involved in the growth of the vessels. Anti-VEGF agents have been in the market for the past 20 years and show significant efficacy in the treatment of neovascular age-related macular degeneration (nAMD); however, some studies show that half of the patients are unresponsive to them. VEGF agents are essential in the survival and maintenance of choroid and retinal pigment epithelium. The loss of polarity and increased secretion of VEGF from the RPE causes pathological changes in CNV. Anti-VEGF drugs are humanized or primates-derived antibodies that either disrupt the structural integrity of the VEGF agents or cause destabilization of the functional efficacy of these molecules [3].

The recognition of VEGF agents in the pathological process of nAMD was recognized in 1983. Since then, multiple studies have been conducted to see the causative role of these agents and target the treatment process. Three famous agents that are widely accepted all over the world for intravitreal injections are bevacizumab, ranibizumab, and aflibercept. These drugs have now revolutionized the management of ocular neovascular pathologies, and newer agents are also being developed to manage the disease better while meeting patients' expectations.

Researchers focus on a better drug delivery system and instigating the use of viruses and genes to develop a long-term drug. There have been some recent successful gene therapy developments, e.g., RGX-314 (Regenxbio, Rockville, MD) and ADVM-022 (Adverum Biotechnologies, Redwood City, CA). These therapies employ non-integrating viral vectors that, in this case, are based on adeno-associated virus (AAV) for transduction of genetic material encoding for anti-VEGF proteins. Patients with wet AMD who have undergone the RGX-314 therapy have received positive results that show up to 85% lowering of treatment burdens for two years or longer with both productions of therapeutic proteins and visual acuity in follow-up examinations. In a similar manner, ADVM-022 delivery by intravitreal injection also has an innovative treatment strategy by treating inflammation through corticosteroids while giving the required anti-VEGF injections less frequently due to positive results in managing clients with inflammation. These are somewhat revolutionary steps in the treatment of AMD wherein the eye itself has the potential to deliver therapeutic proteins and substantially diminish the need for additional treatments [4].

Different physicians employ different regimens and dosing strategies in their respective settings. The treat-and-extend (tex) approach is the most common approach, where the injection is given at each visit, and the visits are adjusted according to the needs of the patients. This approach has become more convenient for patients and healthcare providers because of the cost-effectiveness and fewer visits to the clinic. Another approach is the pro re nata approach, which prescribes the treatment based on anatomical and visual criteria. This approach gives similar visual results to the tex approach but has different results in the long run. The Food and Drug Administration (FDA) approval of ranibizumab and aflibercept for CNV in AMD was given, but bevacizumab is still an off-label drug for the patients. The use of bevacizumab for nAMD has been a debatable topic for physicians all over the world. It is widely used for colon cancer, lung cancer, and breast cancer, but its use in the nAMD has been a controversial topic due to its off-label use and non-approval by FDA. Multiple studies and evidence suggest that the drug is suitable for the treatment of nAMD. However, different studies need to evaluate its long-term use, clinical visits, visual improvements, and comparison with other drugs [5].

Despite the aggressive use of these drugs in the treatment of CNV in AMD, there are still some questions that need further investigation. The comparative efficacy of different anti-VEGF agents, the long-term benefits of the drugs, the cost-effectiveness of different drugs, availability, patient preferences, the availability of drugs in different regions, and the future of these drugs are some of the points that need further review and focus by the researchers. Our literature is focused on data collection regarding using anti-VEGF agents in the CNV of nAMD in recent years. The literature is thoroughly studied and filtered to bring conclusive evidence regarding the progression and future of anti-VEGF agents [6]. 

## Review

Methods

**Figure 1:**
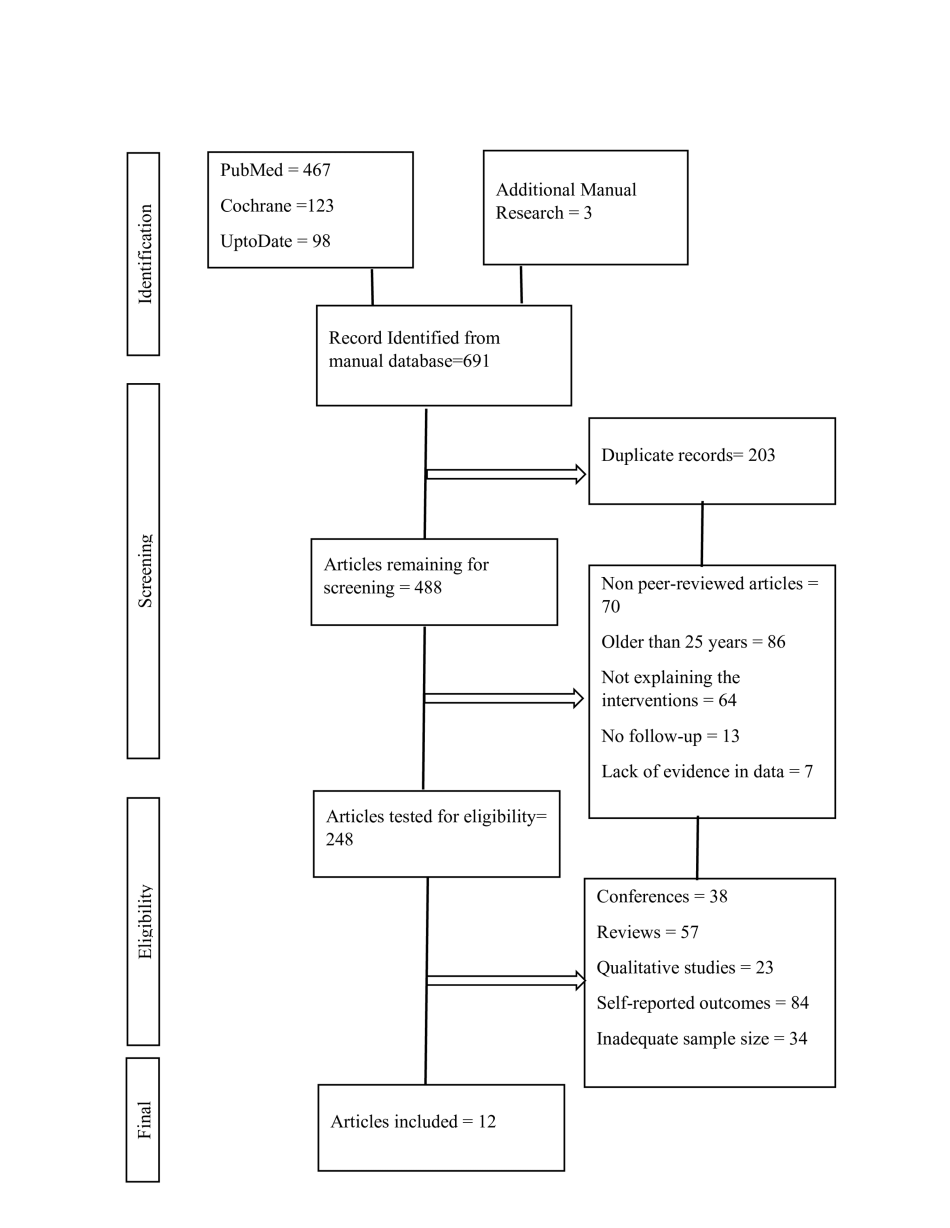
Preferred Reporting Items for Systematic Reviews and Meta-Analyses (PRISMA) diagram of the method adopted to include/exclude studies for the review

A comprehensive literature search was conducted on different academic platforms to yield high-end studies regarding the treatment of nAMD. The search platforms included Google Scholar, Cochrane's database, PubMed, and UptoDate. The keywords used in the search of the study were by the Medical Subject Headings, which included AMD, anti-VEGF therapy for AMD, treatment of nAMD, management of CNV in AMD, comparison of anti-VEGF drugs in AMD, benefits of anti-VEGF therapy, and other related terms. The total number of articles obtained from these resources and some manual research was 691. The articles were reviewed by multiple authors, who removed duplicate studies, which were 203 articles. The remaining articles were screened, and multiple criteria were applied to screen the unimportant articles [7]. The inclusion criteria for the articles were to include the articles published in well-renowned journals, peer-reviewed articles, articles published in the last 25 years, articles with follow-up of at least one year, and articles with evidence backing their conclusions. The exclusion criteria of the review were to exclude articles that were not peer-reviewed, unpublished articles, non-experimental study designs, self-reported outcomes, and inadequate sample size. After being screened, the articles were then scrutinized for the eligibility criteria. The final number of articles included in the study was 12, which were then evaluated for analysis. The summary of the selection process is shared in the Preferred Reporting Items for Systematic Reviews and Meta-Analyses (PRISMA) diagram below (Figure 1). 

Results

The deep study of the abstract and body of the articles revealed various details about them, making them part of this study. The articles in the literature review revealed various details and developments on the role of anti-VEGF therapy in CNV. The first point to be noted is that anti-VEGF therapy only applies to neovascular AMD, also known as wet AMD. As discussed earlier, the pathogenesis of the disease dictates that the VEGF does not play a role in the progression of atrophic macular degeneration. Most studies revealed that all anti-VEGF therapies are clinically equal in improving visual acuity to at least ≥15 Early Treatment Diabetic Retinopathy Scale (ETDRS). Most of the studies in the literature had a more minor scale follow-up of the patients. Only two studies had a longer follow-up of six and 11 years. These studies effectively determined the long-term effects of these drugs on the patients. The oldest study in our review was performed in 2011, and the rest were done in the recent decade. The studies revealed that ranibizumab has superior visual acuity and optical coherence tomography (OCT) outcomes compared to PDT but has similar outcomes to other anti-VEGF agents. Every study in the review used all three known anti-VEGF drugs in their patients and revealed similar outcomes. One study compared the long-term effects of the three anti-VEGF drugs and revealed that all drugs have similar clinical outcomes until one year, but the effect gradually weans off until six years of follow-up. This is due to the non-adherence of patients, increased clinic visits, and cost management for the patient [8]. The study claims that the combinations with fewer clinical visits had better outcomes in the long run. Some studies in the literature compared the cost-effectiveness of the drugs, revealing that bevacizumab was more cost-effective than all the other drugs. The main concern for the patients getting treated with bevacizumab was the off-label use of the drug. It showed that patients showed more concerns about the use of bevacizumab than any other drug, even though it was readily available and had similar clinical outcomes.

Another study was performed on patients aged more than 65 years who were prescribed intravitreal anti-VEGF injections. The study revealed that greater drug adherence was more cost-effective for the patients than no adherence. This is because greater drug adherence had better clinical outcomes, reducing clinic visits and developing other associated complications. Greater adherence with the drug injections was better for the patients in the longer run. One study compared the outcomes of different modes of therapy on the patients. The different modes of dosing and therapies used by clinicians were compared for clinical outcomes, revealing no significant difference in the clinical outcome of the disease. The type of regimen for the patient should be decided based on patient preferences and cost-effectiveness [9].

One of the studies was performed on patients who underwent pars plana vitrectomy, and the benefits of all the anti-VEGF treatments were compared with those of these patients. The study showed that the anti-VEGF agents showed visual improvements and increased anatomical and functional results. This proves that anti-VEGF agents are essential in inhibiting the proliferation of blood vessels in the retina and other eye parts. One retrospective analysis confirmed the requirement for the number of injections for the disease to be completely obliterated. The study compared the number of injections required by the patients of nAMD over a more extended period, which confirmed that bevacizumab requires more injections than aflibercept. This takes us to the point of analyzing the effectiveness of drugs and patients' preferences for their treatment. One study compared the use of different drugs for the patients according to their preferences. The study analyzed the differences in the preferences of patients for drugs in different settings. The clinical results obtained from different drugs were the most probable cause that changed the preference of drugs for patients. Most patients preferred those drugs that gave better visual results than others [10]. The second most common factor influencing their decisions was the cost-effectiveness of the drugs. Patients preferred the drugs that were most cost-effective for them. This decision was the primary focus in the countries with low socioeconomic status. Patients always focus on balancing cost-effectiveness and the best visual results for themselves. This inferred that patients with these diseases always wanted the best available treatments regardless of any other factor. People with low socioeconomic status wanted better treatment in their economic range. The number of clinical visits was the third most common factor in deciding treatment choice. Fewer clinical visits meant lower insurance costs and fewer clinic patient commitments. Most of the studies in our literature were confined to one region, but one study performed by Finger et al. was performed in more than geographical regions with a larger sample size. The research concluded that there was an improvement in the mean lifetime of the patients, and patients had better outcomes in the long run. This study's results can be generalized to other regions of the world (Table 1).

**Table 1:** Summary of the studies included in the literature review BCVA: best corrected visual acuity, ETDRS: Early Treatment Diabetic Retinopathy Scale, NA: not applicable, PPV: pars plana vitrectomy, RCT: randomized control trial, VA: visual acuity

Author	Year	Sample size	Type of study	Type of AMD	Follow-up	Effectivity BCVA ≥ 15 ETDRS	Remarks
Corazza et al. [11]	2021	780	Cohort	Wet	Three to five year	No difference in mean BCVA in long-term.	Long-term study of follow-ups.
Korva-Gurung et al. [12]	2023	827	Cohort	nAMD	Two years	Improvement in 90% eyes.	-
Nunez et al. [13]	2024	45	RCT	Wet	One year follow-up	Similar improvements with different regimens.	Bevacizumab was more cost-effective than ranibizumab
Mulligan et al. [14]	2020	168	Cohort	Wet	One year	No difference in BCVA.	Study done on age >65 years. Adherence is directly related with cost effectiveness.
Mun et al. [15]	2021	44	Retrospective analysis	nAMD	One year	Anatomical improvements in PPV individuals.	Study done on patients who underwent PPV.
Wykoff et al. [16]	2023	226,767	Cohort	nAMD	Six years	Improved VA until one year with loss until six years.	Frequent injections are associated with better outcomes.
Artigas et al. [17]	2019	45	RCT	nAMD	One year	Improved VA similar in all drugs.	-
CATT Research Group et al. [18]	2011	1208	RCT	Wet	One year	Similar improvements.	-
Horner et al. [19]	2021	230	RCT	nAMD	Two year	Increase in BCVA.	Different combination regimens provide similar results.
Finger et al. [20]	2020	3192	Multi-state model cohort	nAMD	11 year	Improvement over a longer follow-up.	Calculated improvement in mean lifetime of the disease.
Cao et al. [21]	2023	106	Retrospective analysis	nAMD	One year	-	Bevacizumab required more injections than aflibercept.
Ozdemir et al. [22]	2022	180	RCT	nAMD	NA	Patient preferred the treatment with the best visual responses with fewer clinic visits.	Treatment preferences.

Some of the adverse effects of the intravitreal injections of anti-VEGF include increased intraocular pressure (IOP), endophthalmitis, retinal detachment, and other similar adverse outcomes. The studies included in our review also analyzed the rate of adverse outcomes in the follow-up of the patients. Studies showed few adverse outcomes in the patients using anti-VEGF drugs. Studies mainly focused on the adverse events of bevacizumab, which is being used as an off-label drug and is one of the most significant concerns of the patients. Multiple studies have shown that the rate of occurrence of adverse reactions in these drugs is similar to each other. Studies show that there is a significant increase in the IOP due to the CNV in AMD rather than the use of the drugs. In fact, in some cases, these drugs have helped in the lowering of IOP by decreasing the CNV. On some occasions, the drugs have helped in the decrease of IOP, which decreased the need for surgical interventions for the management of IOP. There are certain conditions shown in the literature where IOP was increased due to anti-VEGF agents, but the increase was easily manageable by the topical anti-glaucoma drugs. Studies have reported a short-term increase in the IOP after the injection, but it resolves spontaneously and rarely needs any procedure to resolve. Studies have shown variability in the occurrence of the systemic adverse effects of these drugs, which include hypertension, stroke, and thromboembolic events. Some studies claim that there is an increased risk of stroke with ranibizumab than bevacizumab. Some studies claim that bevacizumab is associated with more significant systemic effects than ranibizumab. Some studies claim that systemic adverse effects occur in a similar pattern and ratio in all drugs and patients, showing no difference in the occurrence.

Discussion

The effects of all the famous anti-VEGF drugs have been studied and compared in different studies. The results of all the studies are compiled and evaluated for the newer developments in the field. Most studies have shown a considerable effect of anti-VEGF drugs on improving neovascular age-related macular edema. The disease has no cure, but pharmacological therapy can slow down the progress of the disease. The drugs being used to treat the disease are very costly and without universal availability. The off-label use of bevacizumab is also a debatable question for patients and physicians. Bevacizumab can be an excellent alternative to these costly drugs, but it still requires a great deal of research to qualify for the FDA approval of its use in the retinal disease. A great deal of research and development is still required to devise novel drugs and treatments for the disease. Our literature has exclusively researched and compared the critical points in the studies done in the near decades.

The economic and quality of life analysis of these drugs reveal that they can remarkably improve the quality of life in these patients. The quality of life in these patients depends on improving the vision or decreasing the visual loss to a more significant extent. All these drugs have been shown to improve vision and, thus, the quality of life. The off-label use of bevacizumab can be a significant alternative to other similar but costly drugs. Scientists are also trying to develop better drugs and alternative therapies for these drugs. AMD had a very severe history where most of the patients developed blindness within the three years of the disease. The inculcation of anti-VEGF therapies for nAMD has revolutionized the treatment of this disease. Patients undergoing these therapies have improved their visual acuity rather than avoiding legal blindness. In real-life situations, most patients cannot comply with the treatment process and drop out of the treatment regime. Studies show that more than 50% of the patients drop out of the treatment regime, which decreases their chances of improvement [23,24].

This study has multiple strengths, as it analyzes various kinds of features either related to the disease process or the treatment practices that are going on. The study is focused on various aspects of the conclusive evidence, especially in the recent literature. The inclusion and exclusion criteria have provided a suitable filter for the studies that can help generalize the trends and apply the principles universally. Clear implications of each anti-VEGF drug are analyzed and applied in real-world settings. The balance between patient preference and clinical outcome can be ascertained through this study. The study has revealed that most follow-ups on the studied patients take one year or so. There is a massive lack of extensive follow-up studies that can determine the longer outcomes of these drugs.

There is also much bias in the literature regarding disease development and the treatment prescribed. The studies included in the literature are related to the developed countries. The findings of these studies cannot be generalized over to other diseases. Areas belonging to low socioeconomic backgrounds cannot get access to these treatments, so their data need to be inculcated into the study. The FDA approval of bevacizumab will be a massive breakthrough in disease management due to its cost-effectiveness. Many people are opting for its use despite the off-label use. Most of the studies also lacked data on the anatomical and structural health of the macula after using these drugs, as most of the studies showed improvements only in the clinical aspect of the disease. Studies have also not seen the association between different environmental factors and disease progress. They have not shown data on compliance concerning population distribution and patients' financial conditions. The studies have some inherent bias due to the use of drugs on a specific population as the mean age depicted in the studies. Bevacizumab is used on the population with preserved visual acuity. Different types of CNV in the AMD can also deviate from the results that have not been studied thoroughly. The generalization of results based on studies done at a specific time and area is not applicable in this scenario. Studies should be done by defining the subgroups differently, such as gender, age, area distribution, socioeconomic status, and other related groups. The potential for studies to extrapolate the results in a longer run requires more extended follow-up studies [25].

There is still a massive gap regarding the efficacy of these drugs for more than two years. Most of the studies on AMD have a tiny sample size, which is insufficient to generalize the results. This can be attributed to the fact that most patients drop out of the study after some time and cannot comply with the more extended follow-up studies. There is an excellent need for studies with larger sample sizes and a longer span, which can be helpful in generalizing the results. The patients of AMD vary significantly in age, lifestyle, and underlying comorbidities, which needs better stratification of results. No particular study found in the literature shows the effect of confounding factors on the results of AMD treatment. Most adults suffering from AMD are found with comorbid conditions such as hypertension, diabetes mellitus, or other chronic diseases. Patients can also use different medications related to other diseases, which can lead to changes in the results. Studies involving the role of anti-VEGF can have ethical concerns related to their availability and usage in different areas. Future studies should aim to consider the ethical dilemmas and patient concerns regarding the treatment [26].

There will always be a funding bias when studying treatments and diseases. As the sample size has restrictions, clinical settings also cannot mimic real-life settings; there will always be a bias in the operation of studies and their application in the overall environment. Different therapies must be compared adequately to ascertain the best possible treatment for patients in a particular setting. The relationship between diseases and patients' genetic makeup has not been studied in detail. The reasons for resistance to treatment regimes, recognition of biomarkers, DNA profiling, and other related biochemical elements must be incorporated into the treatment's effectiveness. The scale of measuring the quality of life in the patients usually improves the visual equity, which cannot encompass all the features considered in the quality of life index. There is a need for studies on the patients' perspective that encompasses all the features of patients' quality of life. Environmental factors also influence patients' treatment effectiveness and compliance, and these should be evaluated in the studies. The patients included in the studies are not stratified based on disease progression, which can alter the results during the reporting.

Additionally, the effectiveness of the treatment is measured by assessing the patients' visual improvement. This scale is a subjective experience that varies from patient to patient. Objective assessment of the disease should be done by relying on measurable outcomes, e.g., retinal thickness.

## Conclusions

It has been established through various studies that anti-VEGF therapies are effective in the CNV of AMD. Our review has established some significant developments in the past few years regarding the effectiveness of anti-VEGF drugs. The review can help establish a baseline for future studies. We have also identified some loopholes in the literature regarding the efficacy of anti-VEGF drugs, which must be addressed in future studies. Bevacizumab is being used as an off-label drug in most countries and has not been approved by FDA for retinal usage. It is more cost-effective than other anti-VEGF drugs, but its use is a debatable matter among clinicians. These drugs prevent disease progression, but patient compliance, affordability, and follow-up become significant hurdles in the treatment. The treatment for dry AMD and geographic atrophy (GA) still requires further research and assessment to determine the best treatment options. Other treatment options for AMD, like gene therapy, PDT, and related therapies, are under development, but the higher incidence of the disease poses a significant burden on the health system. Future treatments are focused on long-term therapies that can address the quality of life for the patients, optimize the use of available resources, and decrease the number of visits to the clinic for better compliance. Drug development options in molecular chemistry and genetics have opened newer gates in the development of drugs. Recent progress in gene delivery and targeted extracellular protein degradation techniques may lead to more extraordinary evolution in efficacy, efficiency, and safety and, finally, improvement in patient's quality of life and economic burden.
